# Conotoxin protein classification using free scores of words and support vector machines

**DOI:** 10.1186/1471-2105-12-217

**Published:** 2011-05-29

**Authors:** Nazar Zaki, Stefan Wolfsheimer, Gregory Nuel, Sawsan Khuri

**Affiliations:** 1 Faculty of Information Technology, UAE University, 17551 Al-Ain, UAE; 2 MAP5, University Paris-Descartes, 45 rue des Saints-Peres, Paris, France; 3 Center for Computational Science, University of Miami, USA

## Abstract

**Background:**

Conotoxin has been proven to be effective in drug design and could be used to treat various disorders such as schizophrenia, neuromuscular disorders and chronic pain. With the rapidly growing interest in conotoxin, accurate conotoxin superfamily classification tools are desirable to systematize the increasing number of newly discovered sequences and structures. However, despite the significance and extensive experimental investigations on conotoxin, those tools have not been intensively explored.

**Results:**

In this paper, we propose to consider suboptimal alignments of words with restricted length. We developed a scoring system based on local alignment partition functions, called free score. The scoring system plays the key role in the feature extraction step of support vector machine classification. In the classification of conotoxin proteins, our method, SVM-Freescore, features an improved sensitivity and specificity by approximately 5.864% and 3.76%, respectively, over previously reported methods. For the generalization purpose, SVM-Freescore was also applied to classify superfamilies from curated and high quality database such as ConoServer. The average computed sensitivity and specificity for the superfamily classification were found to be 0.9742 and 0.9917, respectively.

**Conclusions:**

The SVM-Freescore method is shown to be a useful sequence-based analysis tool for functional and structural characterization of conotoxin proteins. The datasets and the software are available at http://faculty.uaeu.ac.ae/nzaki/SVM-Freescore.htm.

## 1 Background

Conotoxins are parts of the neurotoxic peptides isolated from the venom of the marine cone snail of the Genus Conus. They are typically 10-30 amino acids long and contain up to five disulfide bonds [1]. Conotoxins have a variety of action mechanisms, most of which have not yet been sufficiently studied and thus fully understood. However, it appears that many of these peptides modulate the activity of ion channels. The ion channels are key components in a wide diversity of biological processes and are frequent targets in the search for new drugs [2]. Therefore, a conotoxin proven to be effective in drug design has great potential to be used in the treatment of schizophrenia, some neuromuscular disorders, chronic pain, epilepsy, cardiovascular disorders and bladder dysfunction. Assignment of newly sequenced conotoxin into the appropriate superfamily using a computational approach could provide an efficient technique for obtaining or adding valuable preliminary information on the biological and pharmacological functions of these toxins. There are three major classification schemes for conotoxins: gene superfamilies, based on similarities in the translated signal peptide sequence of conotoxin mRNA; cystein framework groups, based on post-translational modifications of the mature conotoxin protein; and pharmacological families, based on relationship between the conotoxin and its molecular target [3]. Thus, there are sixteen superfamilies (A, D, G, I1, I2, I3, J, L, M, O1, O2, O3, P, S, T and Y) [2-8], and within each superfamily there are several groupings according to the presence of two or more disulphide bridges [9]. Conotoxin classification has been recently reviewed and the data is readily available from the ConoServer database [3]. Conotoxins thus provided the ideal protein group to test a new classification algorithm on.

### 1.1 Related methods

Several methods have been suggested for protein homology detection and classification, whereby most of the successful methods were based on profile-sequence or profile-profile alignment. Some of the earlier methods include hidden Markov models (HMM) [10], PSI-BLAST [11,12], COACH [13]and HHsearch [14]. Other methods that utilize structural information are PROSPECT [15], and ProfNet [16]. Profile Comparer [17] is also scoring scheme that aligns profile HMM of protein families and recognizes distance homology relationships well.

In addition, recent years have witnessed remarkable performance enhancement in protein classification stemming from the employment of support vector machines (SVM) as a popular statistical machine learning tool [18,19]. Examples are SVM-Pairwise [20], HMMs combining scores method [21] and profile-profile alignment with SVM [22]. Moreover, several kernel methods such as local alignment kernels [23], profile-based direct kernels [24], SVM-SK [25] and cluster kernels [26] were proposed to develop more powerful remote homology detection methods that eventually assisted in classifying proteins. Furthermore, applying new feature extraction method such as non-negative matrix factorization (NMF), to profile-profile alignment features increased the performance of fold recognition significantly [27].

Despite their high performance, profile-based SVM methods have one essential drawback- an extensive training requirement. To overcome this issue, simpler and more general algorithms have been pursued [28]. A simple comparison process using pairwise protein sequences similarities was suggested in Rankprot [26], in addition to distance-profile methods reported in [29]. The SCOOP approach [30] considered common sequence matches between two Pfam HMM profile search results, and performed better than elaborated methods such as HHsearch in detecting protein superfamily relationship.

Whilst most of the above mentioned methods rely on protein sequence alignment, some researchers turned their attention to classifying conotoxin superfamilies using alignment-free approaches. Mondal et al. [8] used several theoretical approaches for classifying conotoxin proteins into their respective superfamilies based on the primary sequence of the mature conotoxin. They incorporated the concept of pseudo-amino acid composition (PseAAC) [31] to represent peptides in a mathematical framework that includes the sequence-order effect along with conventional amino acid composition. The polarity index attribute - encoding information such as residue surface buriability, polarity, and hydropathy - was utilized to store the sequence-order effect. The representation was further utilized in conjunction with several classifiers such as multi-class SVMs, ISort (Intimate Sorting) predictor [32], least distance algorithms [33,34] and a multiple binary approach [35] - known as the one-versus-rest (1-v-r) SVMs. Another method termed IDQD was recently developed by Hao Lin et al. [9], exploiting a feature extraction approach similar to the Multi-class SVMs. However, a new algorithm of increment of diversity combined with modified Mahalanobis discriminate was used as a classification technique instead of SVM. In this case, the sequence is predicted to be a member of a certain conotoxin superfamily if the corresponding increment of diversity value is the minimum.

### 1.2 Weakness of the recent works

Despite the success of the alignment free methods discussed above, these methods have two major limitations: Firstly, they considered only PseAAC to represent the protein sequence. Conventional amino acid compositions contain 20 components each reflecting the occurrence frequency for one of the 20 native amino acids in a sequence. In contrast, the PseAAC contains additional components that incorporate some sequence-order information via various modes [31]. However, the additional factors attributes were limited to the length of the protein sequence. As most of the conotoxin proteins are typically short (10-30 amino acids long) [36] the PseAAC information is rather limited. With regards to the first 20 attributes which reflect the normalized occurrence frequencies of the 20 native amino acids in the conotoxin protein sequence, short sequence may not reflect statistically valid occurrence frequencies. Secondly, evolutionarily and structural relationships within the conotoxin superfamily were not incorporated. It is well established that homology can be inferred from sequence similarity, and, that homological relationships usually imply the same or at least very similar structural relationships [20,37].

### 1.3 Proposed solution

We set out with the aim of providing a more accurate method of classifying protein sequences, using conotoxins as an example. The ultimate significance of this new method will be in its application to the accurate structure/function classification of protein families important for drug discovery. The work in this paper is motivated by the observation that the pairwise alignment score provides a relevant measure of similarity between protein sequences. The similarity may incorporate biological knowledge about the proteins' evolutionarily structural relationships [23]. However, due to the hyper-variability of mature toxin sequence, similarity methods are often not sensitive enough to indicate all evolutionarily relationships, especially when the homology is weak.

Although many methods have been proposed for protein alignment or comparison, alternative similarity measures are still strongly demanded due to the requirement of fast screening and query in large-scale protein databases [38]. In this study, we introduce an alignment type of feature extraction to represent the proteins. It is based on the idea from the alignment-based method SVM-Pairwise [20]. In SVM-Pairwise method the authors proposed a simple way to represent a protein sequence as a fixed-length vector of real numbers where the resulting vectors can then be used as input to a discriminative learning algorithm. The essential idea was that the interesting characteristics of a protein sequence were effectively captured by measuring how similar protein is to a large collection of other proteins. Therefore, a given protein was compared to every protein in the collection. However, in this case Smith-Waterman scores which was used by Liao et al. [20] to compare two amino acid sequences was replaced by so-called free scores. The underlying model is a "finite temperature" version of local sequence alignment of words of restricted size.

Instead of only focusing on the optimal score (as in the Smith-Waterman algorithm), free scores incorporate possible alternative alignments, similar to the forward score in HMMs. Incorporating possible alternative alignments is particularly important when many independent high scoring regions are expected [39], such as the shifting windows in the feature extraction step (see below in Section 2.1). Hence, we anticipate the free scores to be advantageous.

## 2 Method

The proposed method which we call SVM-Freescore method consists of two major steps:

• Feature extraction: representing each protein sequence by a vector of pairwise similarity scores. The pairwise similarity score is computed using finite temperature word alignment.

• Classification: taking as a kernel the inner product between the feature vector representations to be used in conjunction with SVMs.

In the following sections, we describe the feature extraction step and the classification step.

### 2.1 Feature extraction

Classification using SVM is based on the separation of vectors in an *n *dimensional space by finding hyperplanes. In a first step, it is therefore necessary to represent the objects of interest *X *as so-called feature vectors. This refers to the feature extraction step. The training set *S *is mapped on a *m *× *n *matrix where the rows represent the feature vectors *F^X ^*of the training sequences *X *∈ *S*.

The entries of this matrix are computed as follows. Firstly, we concatenate the database of the training sequences *S *to one long sequence *D *of length ℓ. For example, from *S *= {admn, qghk, il, gedk}, we obtain the sequence *D *= admnqghkilgedk of length ℓ = 14. Secondly, we shift a window of length ℓ*_W _*along *D *such that in each step it is moved by its length. The length of this window determines the dimension *n *of the feature vectors as *n *= ⌈ℓ/ℓ*_W_*⌉, where ⌈*x*⌉ denotes rounding to the next integer larger than or equal to *x*. For the above example, we obtain *n *= 4 for the choice ℓ*_W _*= 4.

Let *W^t ^*denote the *t^th ^*subsequence (*t *= 1 ... *n*) generated by the sliding window, i.e. . In sequence-based feature extraction methods each component of the *F^X ^*is given by a number that measures the similarity between *X *and the *t^th ^*subsequence *W^t^*. This measure can be, for example, the optimal alignment score as in SVM-Pairwise [20] or the so-called free score here.

Note that it is also common practice in bioinformatics research to slide a window by a single position. However, this will generate more subsequences *W^t ^*than simply shifting the window by its size and therefore a significantly larger vector space. For instance, sliding a window of size 4 over *D *yields *n *= ℓ -ℓ*_W _*+ 1 = 11 subsequences, instead of only *n *= 4 as for shifting. Even though the learning ability may depend on the dimension, computational complexity is an essential issue to efficiently handle a large number of protein sequences. Moreover, using a shifting window over the concatenated sequences of the training set may lead to windows consisting of only fragments of the original sequences. This, however, is not a problem as all protein sequences of interest score against the same subsequences. We tested both approaches and the results suggested no significant difference in accuracy.

In the following, we discuss how the actual values of the feature vector were determined.

### 2.2 Finite-temperature word alignment

Our approach is similar to a recently developed method for protein-protein interaction (PPI) using pairwise similarity (PS) [40] which proved to be very powerful. In PPI-PS, the authors employed the Smith-Waterman algorithm [41] to extract the features for a sequence *X*. In this case, each component  of the feature vector is determined by the local alignment score of the sequence *X *against the *t^th ^*subsequence generated by a shifting window.

**Figure 1:**
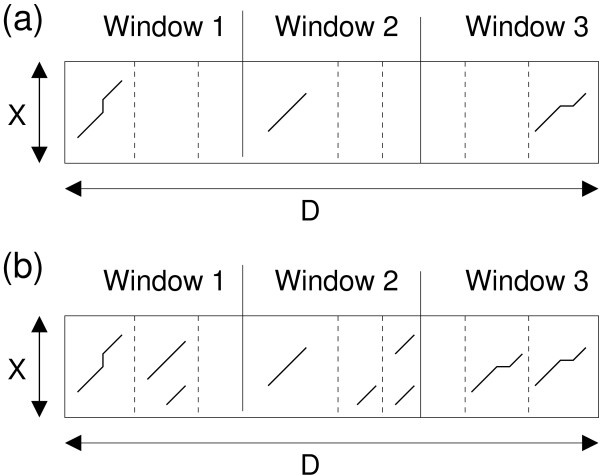
**Optimal alignment vs. finite-temperature alignment**. (a) One highly similar region in the search space (b) Many competitively similar regions in the search space in each window.

The Smith-Waterman algorithm is suitable for problems where one expects one region in the search space with high similarity. Such a situation is shown in Figure 1(a). Apart from slight variations, one alignment with large score dominates in each shifted subsequence. However, in our case this is not exactly the kind of similarity that we wish to measure, for two reasons. Firstly, using a shifting window along a concatenated database may lead to more than one high scoring region within one window due to similarities of the sequence *X *to distinct entries in the original database. Secondly, if the homology is weak there may be distinct nearly-optimal alignments even in the comparison of *X *against one entry in the database. The so-called forward score in HMMs can account for such situations [42]. However, HMMs usually rely on a larger parameter set than score-based alignments. For this reason, we experimented with finite-temperature alignment which is a straight-forward generalization of classical score-based alignment [43,44].

In this framework, we considered not only the optimal alignment but the complete set of possible alignments of the input sequence *X *and *W^t^*. Each alignment  was given an exponential weight  depending on its score  and one free parameter, the "temperature" *T*. The score was computed in the usual way by summing up the entries of the score matrix (here the BLOSUM62) for all aligned positions while taking into account negative contributions for gaps (-11 for open a gap, -1 for extensions). This kind of distribution is called Boltzmann distribution. An efficient algorithm allows us to compute the normalization factor (or *partition function*)

without the need to enumerate all alignments (see below for a variant of this algorithms for word alignments of restricted size). Borrowing terms from statistical physics, we define the *free score *as

These values form the components of the feature vectors in the SVM classifier. In brief, *f *has the following properties:

• For *T *→ 0, *f *equals the optimal score (the optimum is given all the weight)

• For *T *→ ∞, every alignment is given the same weight.

• There is a critical *T_C _*above which the free score growths linearly (instead of logarithmically) with the sequence lengths [39,45-47]. Also the alignment lengths growth unbounded with the length.

This means *T *can be seen as an contrast parameter that allows one to put more or less mass on suboptimal alignments. One should avoid temperatures larger than *T_c _*because related and unrelated sequence pairs can not be distinguished any more and alignments become meaningless.

However, our first experiments showned that the SVM in conjunction with free score works well, but we obtained an unexpected large optimal temperature, even larger than *T_css _*≈ 2.5 [39], where the SVM classification worked best. To understand this better we shuffled the sequences before classification and obtained essentially the same performance. Hence, it is essentially not the order of aligned amino acids that matters, but more the composition of pairs of amino acids in the sequences weighted with an exponential factor like

where *σ *denotes the score matrix and *h_X _*(*a*), *h_W _*(*b*) the frequencies of occurrence of the amino acids *a *and *b *in the sequences.

To avoid giving up the idea of considering many alternative alignments with sequence order effects we restricted the set of possible alignments in the following way. Instead of allowing arbitrary long alignments, only all gapless alignments between pairs of words of maximal length *k*_max _were considered. Let

denote such a pair of aligned words of length *k*. The score of this word is given by , and, the partition function and free score reads as

It is straight forward to formulate a dynamic programming algorithm that computes *Z_T _*in polynomial time. Therefore, let  denote the auxiliary partition function, defined as sum over all words on the subproblem *X*_1 _... *X_i _*and  such that there is a word of length *k *ending at the positions *i *and *j*. This quantities can be computed through the recursion relation

for *i *= 1 ... |*X*|, *j *= 1 ... |*W^t^*| and *k *= 1 ... *k*_max_. The total partition function *Z_T _*is given by . The free score has related limiting properties for *T *→ 0 and *T *→ ∞ as the one for unrestricted alignment, but arbitrary long alignments above *T_C _*become impossible.

We used the free scores in this way in the feature extraction step.

### 2.3 Classification using SVM

Support Vector Machines (SVMs), having strong foundations in statistical learning theory [19], have been successfully applied in numerous areas of computational biology. As shown by Vapnik et al. [18], SVM implements an optimal marginal classifier to minimize the structural risk and offers several associated computational advantages such as the lack of local minima in the optimization. Furthermore, scalability and the generalization capability of SVM [19] make it more suitable for protein classification. To illustrate the idea of using SVM, let us assume that we would like to recognize conotoxin protein sequences belonging to the superfamily "*S_A_*" from a dataset of proteins that contains sequences from various conotoxin superfamilies "non *S_A_*". Let *s *= (*s*_1_, *s*_2_, ... *s_m_*) denote the conotoxin protein sequence of length *m*, where *s_i _*∈ {*A*, *R*, *N*, *D*, *C*, *Q*, *E*, *G*, *H*, *I*, *L*, *K*, *M*, *F*, *P*, *S*, *T*, *W*, *Y*, *V*} and *r *= (*r*_1_, *r*_2_, ..., *r_n_*) denote the input feature vector, where *r_i _*∈ ℜ*^n^*. The classification of the sequence *s *into "*S_A_*" or "non *S_A_*" class finds an optimal mapping from ℜ*^n ^*space into {+1, -1} where +1 and -1 correspond to "*S_A_*" and "non *S_A_*" classes, respectively. Let {(*r_j_*, *q_j_*), *j *= 1, 2, ..., *N*} denote the set of training exemplars, where *q_j _*denotes the desired class ("*S_A_*" or "non *S_A_*") for the input feature vector *r_j _*of sequence *s_j_*; *N *denotes the number of training sequences. SVM first transforms the input to a higher dimensional space with a kernel function and then linearly combines them with a weight vector *w *to obtain the output. In the classification step, SVM constructs a discriminant function by solving the following optimization problem:

Minimize(1)

subject to the constrains(2)

where slack variables *ξ_j _*represent the magnitude of the classification error, *ϕ *represents the mapping function to a higher dimension *n*, *b *is the bias used to classify the protein samples and *C*(> 0) is the regularization parameter that decides the trade-off between the training error and the margin of separation [18]. The minimization of the above optimization problem is equivalent to maximizing the following quadratic function:(3)

subject to 0 ≤ *α_j _*≤ *C *and .

The function *K*(*r_j_*, *r_i_*) in this case is called the kernel function.

Once the parameters *α_j _*are obtained from the optimization, the resulting discriminant function *f *is given by(4)

where bias *b *is chosen so that *q_j_f*(*r_j_*) = 1 for all *j *with 0 <*α_j _*<*C*. The class corresponding to the input pattern *r_i _*is "*S_A_*" if *f *(*r_i_*) > 0 or "non *S_A_*" if *f*(*r_i_*) < 0.

In this study, the Radial Basis Function (RBF) kernel was employed which is formulated as follows:(5)

where *γ*(> 0) is the scaling parameter. The RBF kernel non-linearly maps samples into a higher dimensional space, therefore, unlike the linear kernel, it can handle the case when the relation between class labels and attributes is nonlinear.

### 2.4 Datasets

The evaluation of the SVM-Freescore method is based on two datasets. The first dataset was developed by Mondal et al. [8] and it will be referred to in this paper as "DATASET-1". The conotoxin sequences were collected from the Swiss-Prot release 47.1 [48]. Superfamilies with a few sequences such as P-conotoxin and S-conotoxin were not included in the analysis. I-conotoxin superfamily was not included either as it was previously divided into two distinct gene superfamilies, namely I1-conotoxin and I2-conotoxin. The outcome of this process was a dataset that includes 156 mature conotoxin sequences from A (*S_A_*), M (*S_M_*), O (*S_O_*) and T (*S_T _*) superfamilies. The mature peptide sequence is often far less conserved than the signal sequences [49]. Data redundancy was removed using a greedy incremental algorithm [50] as implemented in CD-HIT program (CD-HIT is a program for clustering large protein database at high sequence identity threshold). The final dataset consists of 116 entries from four conotoxin superfamilies. A negative dataset N (*S_N_*) including sequences that do not belong to any of the four aforementioned superfamilies was formed from different eukaryotes with diverse functions. The CD-HIT program was used once again to screen the negative set which was resulted in 60 sequences with sequence identity lesser than 40%.

According to the sequences' experimental annotations, the 116 sequences can be divided into four subsets, for each superfamily. The following is the partition of the overall set *S*:(6)

where *S*^non-tox ^and *S*^tox ^are the sets containing all non-conotoxin and conotoxin sequences respectively. The numbers of proteins thus obtained for the four subsets are given in Table 1.

**Table 1:** Number of the conotoxin protein examples in each of the four subsets.

Subset	Superfamily	No. of Sequences
*S_A_*	A-conotoxin	25
*S_M_*	M-conotoxin	13
*S_O_*	O-conotoxin	61
*S_T_*	T-conotoxin	17
*S^tox^*		116

For generalization purpose it was necessary to evaluate our method based on curated and high quality database. As far as we are aware, ConoServer http://www.conoserver.org is the only public database that specializes in conopeptide sequences and three-dimensional structures [3]. ConoServer provides up-to-date information on the sixteen known gene superfamilies. The majority of the sequences and structures found in ConoServer are associated with peer reviewed articles [3]. As of March 2011, ConoServer contained data for 3660 conopeptide sequences. Only complete precursor sequences from which the mature peptide was also isolated at the protein level were retrieved. The unambiguous identification of the gene superfamily requires the complete precursor sequence. Superfamilies with insignificant number of sequences (< 15) were excluded from this study (G, I3, J, L, P, S and Y). Sequences which contain unknown amino acids were also excluded. Finally, we obtained a dataset containing 858 sequences from nine Superfamilies. The numbers of protein sequences thus obtained are given in Table 2. The final dataset will be referred to in this paper as "DATASET-2".

**Table 2:** Number of the conotoxin protein examples in each of the nine subsets.

Subset	Superfamily	No. of Sequences
*S_A_*	A-conotoxin	201
*S*_*I*1_	I1-conotoxin	32
*S*_*I*2_	I2-conotoxin	34
*S_M_*	M-conotoxin	86
*S*_*O*1_	O1-conotoxin	318
*S*_*O*2_	O2-conotoxin	41
*S*_*O*3_	O3-conotoxin	19
*S_D_*	D-conotoxin	18
*S_T_*	T-conotoxin	109
*S^tox^*		858

Once the benchmark datasets DATASET-1 and DATASET-2 were constructed, the subsequent problem is how to find an effective prediction engine to represent the protein samples for training them and conducting the predictions.

## 3 Results

In this section, we investigate the ability of the proposed SVM-Freescore method to classify conotoxin superfamilies.

In our first experimental work, we tested the performance of SVM-Freescore on DATASET-1. A jackknife cross validation test was used since it is deemed the most rigorous among others and hence it has been widely adopted by researchers [8,9,51]. The performance of SVM-Freescore was measured by how well the system can recognize members of any of the conotoxin superfamilies. In order to analyze the evaluation measures, we first explain the contingency table as shown in Table 3. The entries of the four cells of the contingency table are described as follows:

**Table 3:** The contingency table.

	Related sequences	Unrelated sequences
Sequence classified related	True positives (*tp*)	False negatives (*fn*)
Sequence classified unrelated	False positives (*fp*)	True negatives (*tn*)

• *tp*: related conotoxin protein sequences classified as "related".

• *fn*: unrelated conotoxin protein sequences classified as "related".

• *fp*: related conotoxin protein sequences classified as "unrelated".

• *tn*: unrelated conotoxin protein sequences classified as "unrelated".

• *all*: total number of conotoxin protein sequences.

The information encoded in the contingency table was used to calculate the following evaluation measures: Sensitivity (SN) = *tp*/(*tp *+ *fn*), Specificity (SP) = *tn*/(*tn *+ *fp*) and Accuracy (AC) = (*tp *+ *tn*)/*all*.

Following the procedure used in jackknife cross-validation test, we analyzed the behavior and described the ability of the SVM-Freescore to compute the similarity among conotoxin protein sequences. The objective of the experiments was to observe the influence of varying the tunable parameters of the temperature (*T*), maximum word size (*k*_max_) and the shifting window size (ℓ*_W _*) in the classification system. These parameters are introduced in Section 2. Recall that ℓ*_W _*is related to the dimension of the feature vector space. As for the SVM parameters, the kernel scaling parameter *γ *was set to 0.04 and the penalty parameter *C *was set to 100. The training and testing attributes were linearly scaled to the range between -1 and +1 prior to applying the SVM. The main advantage of the scaling is to avoid attributes in greater numeric ranges dominate those in smaller numeric ranges [52]. In this case, we employed the Library for Support Vector Machines [53] available at http://www.csie.ntu.edu.tw/~cjlin/libsvm to classify the contoxin proteins.

### 3.1 Effectiveness of varying temperature parameter *T*

In this set of experiments, we analyzed the effect of varying the temperature parameter *T*, on the generalization performance of the SVM-Freescore learner that manipulates the feature extraction step. A series of experiments was conducted based on DATASET-1 to study the performance of the SVM-Freescore by widely varying *T*. We describe the results of these experiments in Table 4, where the relationship between different values of *T *and the corresponding influence of the classification accuracy (AC) percentage on A, M, O and T conotoxin suberfamilies are shown. The maximum word size *k*_max _and the shifting window size ℓ*_W _*were both set to 2 and 100, respectively. A temperature of 3 was observed to generate the best average optimal results of 92.898%.

**Table 4:** Effectiveness of varying temperature parameter *T*.

*T*	A	M	O	T	Average
1	85.8	92.61	47.73	90.34	79.12
2	92.05	92.61	90.91	95.45	92.755
3	93.75	93.18	88.07	96.59	92.898
4	93.18	91.48	86.36	96.59	91.903
5	91.48	92.05	86.93	94.32	91.195
6	90.91	92.05	87.5	93.75	91.053
7	91.48	92.61	86.93	93.75	91.193
8	90.34	92.61	87.5	93.75	91.05
9	90.34	92.61	87.5	93.75	91.05
10	90.34	92.61	87.5	93.75	91.05

### 3.2 Effectiveness of varying the maximum word size *k*_max_

One of the important parameters needed to tune the system performance is the maximum word size *k*_max_. In this set of experiments, we analyzed the effect of varying the maximum word size *k*_max_. A series of experiments was conducted to study the performance of the SVM-Freescore by varying *k*_max_. We describe the results of these experiments in Table 5, where the relation between different values of *k*_max _and the corresponding influence of the classification accuracy on A, M, O, T conotoxin suberfamilies are shown. The temperature parameter *T *and the shifting window size ℓ*_W _*were both set to 3 and 100, respectively. A word alignment parameter value of 4 was observed to generate the best average optimal results of 93.323%.

**Table 5:** Effectiveness of varying word parameter *k*_max_.

*k*_max_	A	M	O	T	Average
1	85.8	92.61	81.82	91.48	87.928
2	92.05	92.61	90.91	95.45	92.755
3	92.61	92.05	90.91	93.18	92.188
4	96.02	92.61	94.89	89.77	93.323
5	90.34	89.2	97.73	89.77	91.76

### 3.3 Effectiveness of varying window size ℓ*_W_*

In this experimental work we studied the effect of varying the window size ℓ*_W_*. We kept the values of the parameters *T *and *k*_max _fixed to 3 and 4, respectively, and learn the classifier for different values of ℓ*_W_*. The results of this set of experiments are given in Table 6, where the relation between different values of *n *and the corresponding influence of the classification accuracy on A, M, O, T conotoxin superfamilies are shown. From these results, we find out that the performance of the SVM-Freescore varies with varying window size *n *and peaks at a value of 300.

**Table 6:** Effectiveness of varying window size ℓ_*W*_.

ℓ*_W_*	A	M	O	T	Average
10	86.36	93.75	73.3	65.91	79.83
20	93.75	94.89	94.89	89.77	93.325
30	96.59	97.16	93.75	94.89	95.5975
40	96.59	98.3	93.75	96.02	96.165
50	97.16	97.73	93.18	96.59	96.165
60	96.59	98.86	94.32	95.45	96.305
70	97.73	99.43	94.32	96.59	97.0175
80	97.16	98.86	93.75	93.75	95.88
90	97.16	98.3	95.45	94.89	96.45
100	96.59	99.43	94.32	95.45	96.4475
200	96.02	98.3	98.3	94.32	96.735
300	99.43	98.86	99.43	99.43	99.29
400	97.73	98.3	95.45	96.02	96.875
500	97.16	96.59	96.02	93.75	95.88
600	96.59	94.32	96.02	94.32	95.3125
700	95.45	93.18	93.75	96.02	94.6
800	94.89	91.48	95.45	93.75	93.8925
900	95.45	94.32	95.45	96.02	95.31
1000	95.45	90.91	93.18	94.89	93.6075

### 3.4 SVM-Freescore performance evaluation

To evaluate the performance of the SVM-Freescore approach, the jackknife test was used. The temperature parameter *T*, the maximum word size *k*_max_, and the window size ℓ*_W _*were set to 3, 4 and 300 respectively. When applied on DATASET-1 and DATASET-2, the proposed method was able to achieve remarkable AC, SN, SP and ROC accuracy as listed in Table 7 and Table 8. The ROC is the fraction of the true positives (TPR = true positive rate) vs. the fraction of false positives (FPR = false positive rate).

**Table 7:** Overall results based on DATASET-1.

Conotoxin Superfamily	AC	SN	SP	ROC	10-fold Cross- Validation
A	0.9943	0.96	1	0.9925	0.983
M	0.9886	0.9836	1	0.9976	0.9773
O	0.9943	0.9836	1	0.9998	0.9772
T	0.9943	1	0.987	1	0.9943

**Table 8:** Overall results based on DATASET-2.

Conotoxin Superfamily	AC	SN	SP	ROC	10-fold Cross- Validation
A	0.9811	0.985	0.9787	0.9981	1
I1	0.9943	0.9375	0.998	0.9937	0.9943
I2	0.9925	0.9412	0.996	0.9995	1
M	0.9830	0.9535	0.9887	0.9976	0.9659
O1	0.9906	0.9937	0.9858	0.9998	1
O2	0.9943	0.9756	0.9959	0.9996	0.9943
O3	1	1	1	1	1
D	1	1	1	1	1
T	0.9811	0.9541	0.9881	0.9932	.9773

The method was also tested using *μ*-fold cross-validation, we first divided the training set into *μ *subsets of equal size. Sequentially one subset was tested using the classifier trained on the remaining *μ *- 1 subsets. Thus, each instance of the whole training set was predicted once, so the cross-validation accuracy was the percentage of data which were correctly classified. In Table 7 and Table 8, we listed 10-fold cross-validation results based on DATASET-1 and DATASET-2 respectively.

## 4 Discussion

The BLAST algorithm was tested by Mondal et al. [8] to scan against the non-redundant Swiss-Prot database containing 202,310 sequences. The accuracy values for identifying the members of A, M, O and T superfamilies were 88.0%, 69.2%, 85.2% and 11.8% respectively. Thus, it can be interpreted from the performance that the BLASTP tool for searching homologues is not suitable for the hyper variable conotoxins. Therefore, it was imperative to use a superior classification system.

In Table 9, we further compared the performance of the SVM-Freescore to several other methods such as IDQD, multi-class SVMs, One-versus-rest SVMs, Least Hamming distance and ISort predictor to classify *S_A_*, *S_M_*, *S_O_*, *S_T _*and *S_N _*subsets of peptides. Table 9 shows that SVM-Freescore was able to add considerable accuracy.

**Table 9:** A performance comparison of the SVM-Freescore and other existing methods.

Method	A	M	O	T
	SN (SP)	SN (SP)	SN (SP)	SN (SP)
SVM-Freescore	0.960 (1.000)	0.984 (1.000)	0.984 (1.000)	1.000 (0.987)
IDQD	0.960 (0.923)	0.923 (1.000)	0.820 (0.893)	0.940 (0.940)
Multi-class SVMs	0.840 (0.955)	0.920 (0.800)	0.870 (0.869)	0.940 (0.940)
One-versus-rest SVMs	0.840 (0.955)	0.846 (1.000)	0.820 (0.962)	0.765 (0.929)
Least Hamming distance	0.800 (0.667)	0.539 (0.539)	0.771 (0.723)	0.824 (0.824)
ISort	0.760 (0.792)	0.692 (0.600)	0.705 (0.683)	0.882 (0.790)

**Figure 2:**
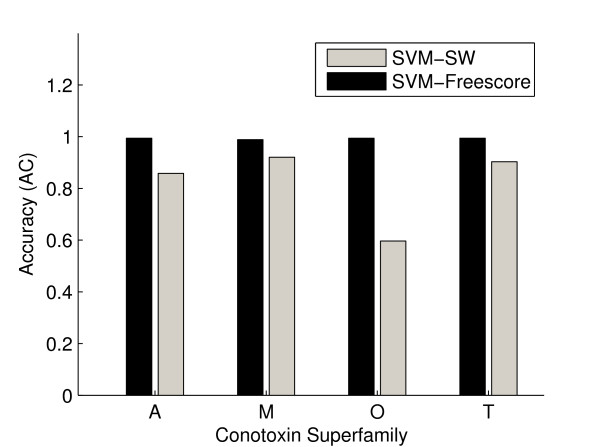
**A performance comparison using the traditional Smith-Waterman alignment in conjunction with SVM (SVM-SW) and the SVM-Freescore**.

A performance comparison using the traditional Smith-Waterman alignment in conjunction with SVM and the SVM-Freescore is also shown in Figure 2. Default Smith-Waterman alignment parameters were used; gap opening penalty and extension penalties of 11 and 1, respectively, and the BLOSUM 62 matrix. The window size ℓ*_W _*was set to 300. The results shown in Figure 2, indicate significant accuracy improvement when the traditional Smith-Waterman alignment has been replaced with the model of finite temperature word alignment.

The fact that our SVM-Freescore algorithm was able to classify the conotoxin dataset into its gene superfamilies shows that there is enough information in the amino acids sequences collected to divide them into biologically relevant groupings. Conotoxin has been proven to be effective in drug design and could be used to treat various disorders. SVM-Freescore can therefore be used to assign conotoxin proteins found, for example, in newly annotated genomes, into their correct superfamily.

## 5 Conclusion

In this paper, we introduced a new representation for the sample of conotoxin protein by incorporating its evolution information using an influential mean of pairwise sequence comparison. We considered finite temperature alignment of words as a technique for protein feature extraction and representation. This approach was motivated by the observation that using a shifting window may lead to distinct alternative alignments with large scores. However, when we let the possible alignments be unrestricted, only the composition of pairs of letters seemed to be relevant in the parameter range were the SVM works best. To account for sequence order effects, at least up to short lengths, we restricted the length of allowed alignments. The extracted features were then used in conjunction with SVM to discriminate between different conotoxin superfamilies. The proposed method demonstrated an improved sensitivity and specificity when compared to other conotoxin classification methods, and is therefore a useful sequence-based analysis tool for protein the classification of protein groups such as conotoxins.

To further improve the prediction quality, it is necessary to incorporate further biological evidence such as gene ontology, protein-protein interaction and inter-domain linker regions knowledge.

## Authors' contributions

NZ contributed to the conceptual development of SVM-Freescore, designed and performed the experimental work and the statistical analysis, drafted the manuscript. SW and GN developed and implemented the free score part of the method. SK contributed to the biology concepts presented in the paper. All authors read and approved the final manuscript.
